# Supplementary material for: A small-molecule SARS-CoV-2 inhibitor targeting the membrane protein
Source: Nature. 2025 Mar 26;640(8058):506–13. doi: 10.1038/s41586-025-08651-6 (PMC11981937; doi:10.1038/s41586-025-08651-6)
Supplement: Supplementary file 1 — Supplementary Methods, Supplementary Table 1 and Supplementary References. [file 41586_2025_8651_MOESM1_ESM.pdf]

nature portfolio

https://doi.org/10.1038/s41586-025-08651-6

Supplementary information

A small-molecule SARS-CoV-2 inhibitor targeting the membrane protein

In the format provided by the authors and unedited

Supplementary Methods

Synthesis of JNJ-9676: General information

Reactions were performed in air or, when oxygen- or moisture-sensitive reagents or intermediates were employed, under an inert atmosphere (nitrogen or argon). When appropriate, reaction apparatuses were dried under dynamic vacuum using a heat gun and anhydrous solvents (Sure-Seal™ products from Aldrich Chemical Company, Milwaukee, Wisconsin or DriSolv™ products from EMO Chemicals, Gibbstown, NJ) were employed. Other commercial solvents and reagents were used without further purification. Products were generally dried under vacuum before being carried on to further reactions or submitted for biological testing. Unless otherwise noted, chemical reactions were performed at room temperature (about 23 degrees Celsius).

Unless noted otherwise, all reactants were obtained commercially and used without further purification or were prepared using methods known in the literature.

Abbreviations used are: aq, aqueous; rt, room temperature; h, hours; min, minutes; CO, carbon monoxide; $CDCl_3$, deuterochloroform, DBAD, di-*tert*-butyl azodicarboxylate; DMSO, dimethylsulfoxide; DMF, dimethylformamide; DCM, dichloromethane; DPPF, 1,1'-bis(diphenylphosphino)ferrocene; Et_3N, triethylamine; EtOAc, ethyl acetate; EtOH, ethanol; ESI, electrospray ionization; MeOH, methanol; PE, petroleum ether; $Pd(OAc)_2$, palladium(II) acetate; PPh_3, triphenylphosphine; Ph_3PO, triphenylphosphine oxide ; MTBE, *tert*-butyl methyl ether; THF, tetrahydrofuran; br, broad; °C, degrees Celsius; d, doublet; dd, doublet of doublets; g, gram; Hz, hertz; M, molar; m, multiplet; mg, milligram; MHz, megahertz; mL, milliliter, µL, microliter, mmol, millimole; s, singlet.

Nuclear Magnetic Resonance (NMR): ^{1}H NMR spectra were recorded on a Bruker DPX-400 spectrometer with standard pulse sequences, operating at 400 MHz. Chemical shifts (δ) are

reported in parts per million (ppm) downfield from tetramethylsilane (TMS), which was used as internal standard.

Synthesis scheme of JNJ-9676

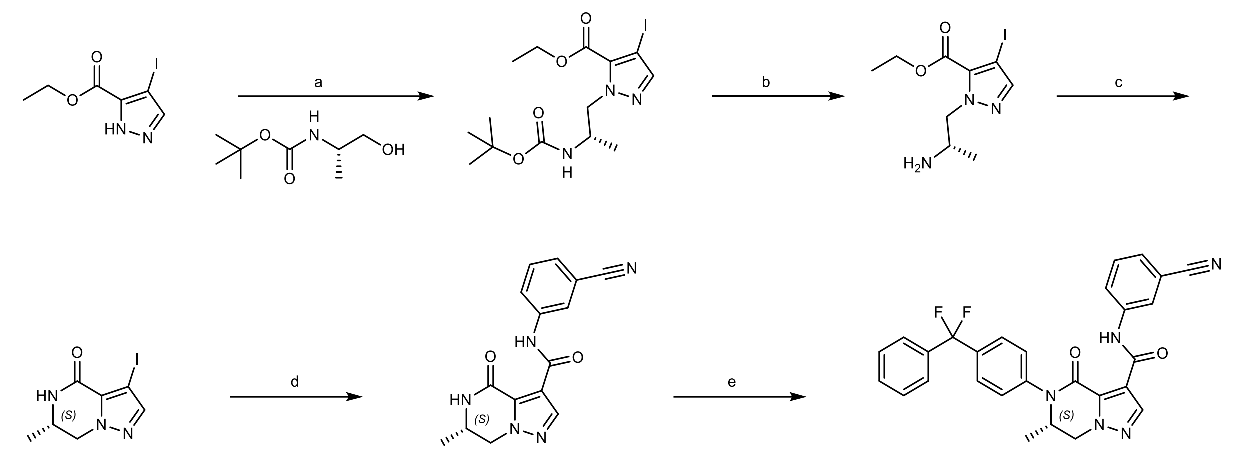

Synthesis scheme of JNJ-9676 a: PPh_3, DBAD, THF, rt, 16 h, quant. **b:** 4M HCl/1,4-dioxane, CH_3CN, rt, 16 h, 76% **c:** $NaHCO_3$, H_2O, rt, 16 h, 56% **d:** 3-Aminobenzonitrile, Et_3N, $Pd(OAc)_2$, DPPF, 1,4-dioxane, CO (1.2 atm), 90°C, 16 h, 95% **e:** 1-Bromo-4-(difluoro(phenyl)methyl)benzene, N^1,N^2-dimethylethane-1,2-diamine, K_2CO_3, CuI, DMF, toluene, 100°C, 12 h, 37%.

Synthesis procedure of JNJ-9676

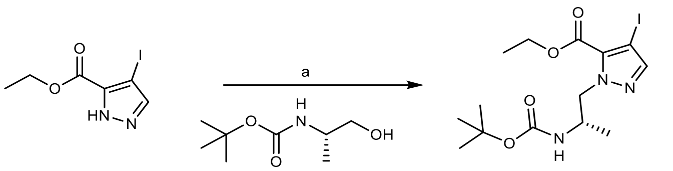

A solution of ethyl 4-iodo-1H-pyrazole-5-carboxylate **1** (42.0 g, 157.9 mmol), *tert*-butyl (S)-(1-hydroxypropan-2-yl)carbamate **2** (55.3 g, 315.7 mmol), Ph_3P (74.5 g, 284.2 mmol) and DBAD (65.4 g, 284.2 mmol) in THF (800 mL) was stirred at room temperature and under nitrogen for 16 h. The mixture was concentrated under reduced pressure. MTBE (600 mL) was added and the mixture stirred at room temperature for 1 h. Then, the mixture was filtered to remove Ph_3PO and

concentrated under reduced pressure. The residue was purified by silica gel chromatography (20%-25%, EtOAc/PE) to afford ethyl (S)-1-(2-((*tert*-butoxycarbonyl)amino)propyl)-4-iodo-1H-pyrazole-5-carboxylate **3** (117.6 g, quant.) as a colorless oily solid, that was used as such in the next reaction. ^1H NMR (400 MHz, $CDCl_3$) δ: 7.58 (s, 1H), 6.25 (br s, 2H), 4.81 (br d, *J* = 6.8 Hz, 1H), 4.62 - 4.56 (m, 2H), 4.19 - 4.08 (m, 1H), 1.35 (s, 12H), 1.13 (d, *J* = 6.8 Hz, 3H). Mass spectrum (ESI, m/z): calcd. for $C_{14}H_{22}IN_3O_4$, 423.1; found $[M+H]^+$, 424.0.

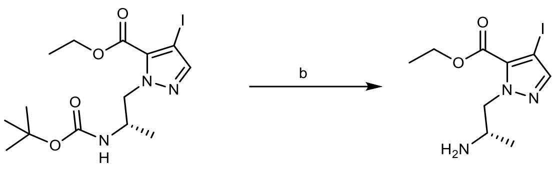

HCl/dioxane (570 mL, 2.28 mol, 4 M) was added to a stirred mixture of ethyl (S)-1-(2-((*tert*-butoxycarbonyl)amino)propyl)-4-iodo-1H-pyrazole-5-carboxylate **3** (117.4 g, 277.4 mmol) in CH_3CN (570 mL). The mixture was stirred at room temperature for 16 h. Then, the mixture was filtered, and the filtered cake was washed with EtOAc and MTBE. The residue was dried under vacuum to afford ethyl (S)-1-(2-aminopropyl)-4-iodo-1H-pyrazole-5-carboxylate hydrochloride **4** (69.1 g, 76% yield) as a white solid, that was used as such in the next reaction. Mass spectrum (ESI, m/z): calcd. for $C_9H_{14}IN_3O_2$, 323.0; found $[M+H]^+$, 324.1.

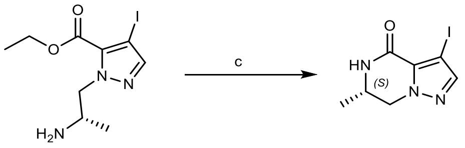

$NaHCO_3$ (72.5 g, 863.5 mmol) was added to a stirred mixture of ethyl (S)-1-(2-aminopropyl)-4-iodo-1H-pyrazole-5-carboxylate hydrochloride **4** (69 g, 191.9 mmol) in H_2O (2 L). The mixture was stirred at room temperature 16 h. The reaction mixture was filtered under vacuum. The filtered cake was washed with H_2O and PE. The residue was dried under reduced pressure to afford (S)-3-iodo-6-methyl-6,7-dihydropyrazolo[1,5-a]pyrazin-4(5H)-one **5** (30.6 g, 56% yield)

as a white solid. Part of this product (10.5 g, 37.9 mmol) was dissolved in H_2O (250 mL) and the mixture was stirred at room temperature 16 h. The reaction mixture was filtered under vacuum. The filtered cake was washed with H_2O and PE. The residue was dried under reduced pressure to afford (S)-3-iodo-6-methyl-6,7-dihydropyrazolo[1,5-a]pyrazin-4(5H)-one **5** (10.1 g) as a white solid, that was used in the next reaction. ^{1}H NMR (400 MHz, DMSO-d_6) δ: 8.32 (s, 1H), 7.67 (s, 1H), 4.43 (dd, J = 3.6, 11.6 Hz, 1H), 4.05 - 3.92 (m, 2H), 1.20 (d, J = 6.4 Hz, 3H). Mass spectrum (ESI, m/z): calcd. for $C_7H_8IN_3O$, 277.0; found $[M+H]^+$, 278.0.

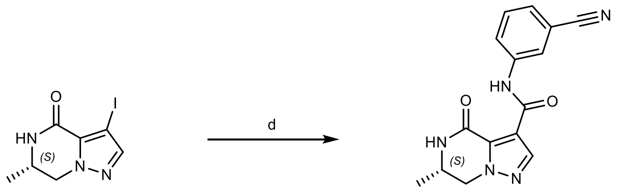

A mixture of (S)-3-iodo-6-methyl-6,7-dihydropyrazolo[1,5-a]pyrazin-4(5H)-one **5** (20.0 g, 72.2 mmol), 3-aminobenzonitrile (25.6 g, 216.6 mmol), Et_3N (30.1 mL, 217 mol), $Pd(OAc)_2$ (405 mg, 1.8 mmol) and DPPF (1.60 g, 2.89 mmol) in 1,4-dioxane (500 mL) was stirred at 90°C under CO atmosphere (1.2 atm) for 16 h. The mixture was treated with HCl (500 mL, 1M) and water. The suspension was filtered and the solid was washed with HCl (500 mL, 0.5M), water (250 mL), methanol (250 mL), EtOAc (250 mL) and MTBE (500 mL) to afford (S)-N-(3-cyanophenyl)-6-methyl-4-oxo-4,5,6,7-tetrahydropyrazolo[1,5-a]pyrazine-3-carboxamide **6** (20.5 g, 95% yield) as a white solid. ^{1}H NMR (400 MHz, DMSO-d_6) δ: 12.86 (s, 1H), 9.26 (s, 1H), 8.19 (s, 1H), 8.12 (s, 1H), 7.76 (br d, J = 7.6 Hz, 1H), 7.63 - 7.49 (m, 2H), 4.55 (br d, J = 8.8 Hz, 1H), 4.21 - 4.05 (m, 2H), 1.28 (d, J = 6.0 Hz, 3H). Mass spectrum (ESI, m/z): calcd. for $C_{15}H_{13}N_5O_2$, 295.1; found $[M+H]^+$, 296.1.

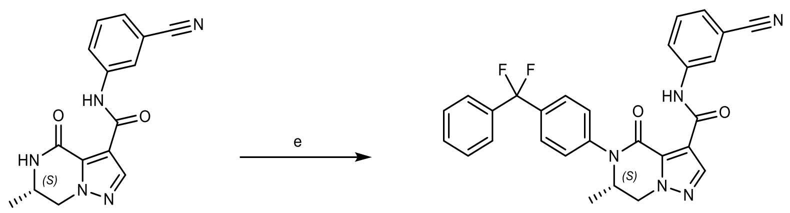

1-Bromo-4-(difluoro(phenyl)methyl)benzene (182 mg, 0.64 mmol), N^1,N^2-dimethylethane-1,2-diamine (17 mg, 0.19 mmol), K_2CO_3 (133 mg, 1 mmol) and CuI (36.8 mg, 0.2 mmol) were added to a stirred solution of (S)-N-(3-cyanophenyl)-6-methyl-4-oxo-4,5,6,7-tetrahydropyrazolo[1,5-a]pyrazine-3-carboxamide **6** (95 mg, 0.3 mmol) in DMF (0.5 mL, dried with 3Å molecular sieves) and toluene (5 mL, dried with 3Å molecular sieves) under nitrogen. The mixture was stirred at 100°C for 12 h. The reaction was monitored by LC-MS. The cooled mixture was filtered and washed with EtOAc. The filtrate was diluted with water and extracted with additional EtOAc. The combined organic layers were washed with brine, dried over Na_2SO_4, filtered and concentrated under reduced pressure. The crude product was purified by silica gel chromatography (50-100% EtOAc/PE) to afford (*S*)-N-(3-cyanophenyl)-5-(4-(difluoro(phenyl)methyl)phenyl)-6-methyl-4-oxo-4,5,6,7-tetrahydropyrazolo[1,5-a]pyrazine-3-carboxamide JNJ-9676, that was freeze-dried to afford a light yellow solid (61 mg, 37% yield). HRMS (ESI, m/z): $[M+H]^+$ Calcd. for $C_{28}H_{22}F_2N_5O_2$, 498.1736, found 498.1747.

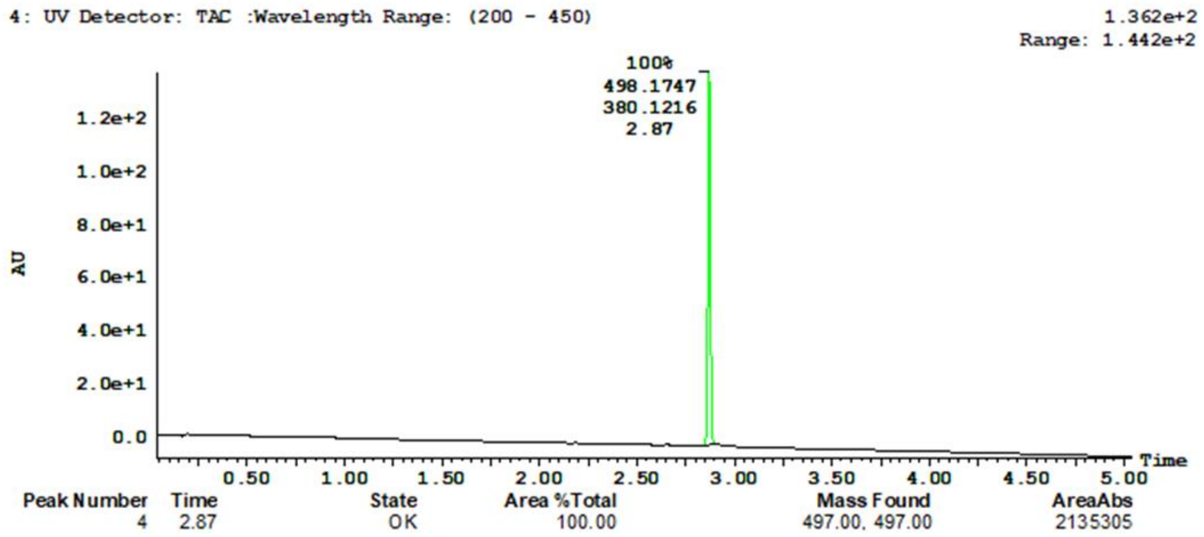

A sample for acquiring NMR spectra was dissolved in DMSO-d6 containing TMS as an internal reference for 1H chemical shifts. 1D ^{1}H, 1D ^{13}C, 2D ^{1}H-^{1}H COSY, 2D ^{1}H-^{13}C HSQC, and 2D ^{1}H-^{13}C HMBC NMR spectra were recorded on a Bruker Avance-500 spectrometer operating at a proton frequency of 500.13 MHz, a carbon frequency of 125.76 MHz and 298 K temperature. The data was processed using the Bruker TOPSPIN program v4.1, and ^{1}H and ^{13}C chemical shifts were analyzed using ACD/Spectrus software 2023 v1.1.

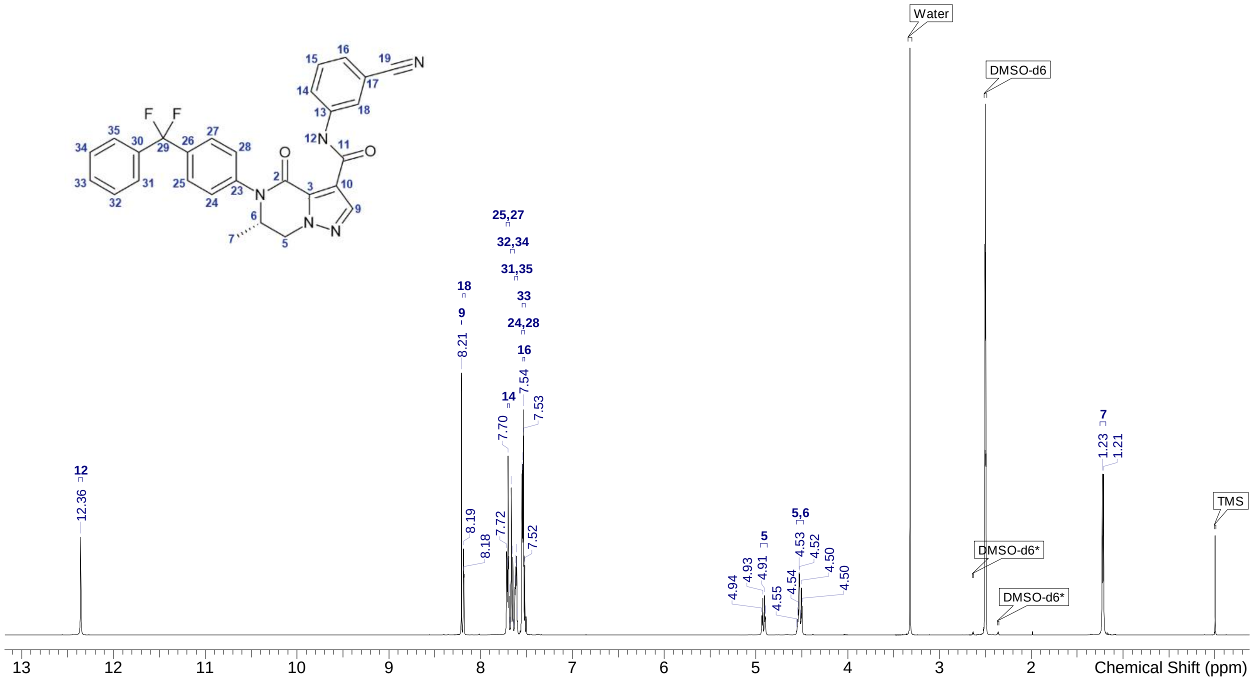

^{1}H NMR (500.13 MHz, DMSO-d_6) δ ppm 12.36 (s, 1 H) 8.21 (s, 1 H) 8.17 - 8.19 (m, 1 H) 7.68 - 7.72 (m, 2 H) 7.68 - 7.71 (m, 1 H) 7.64 - 7.68 (m, 2 H) 7.60 - 7.63 (m, 2 H) 7.52 - 7.55 (m, 2 H) 7.52 - 7.54 (m, 1 H) 7.52 (s, 2 H) 4.88 - 4.95 (m, 1 H) 4.48 - 4.56 (m, 2 H) 2.36 - 2.37 (m, 1 H) 1.22 (d, *J*=6.6 Hz, 3 H)

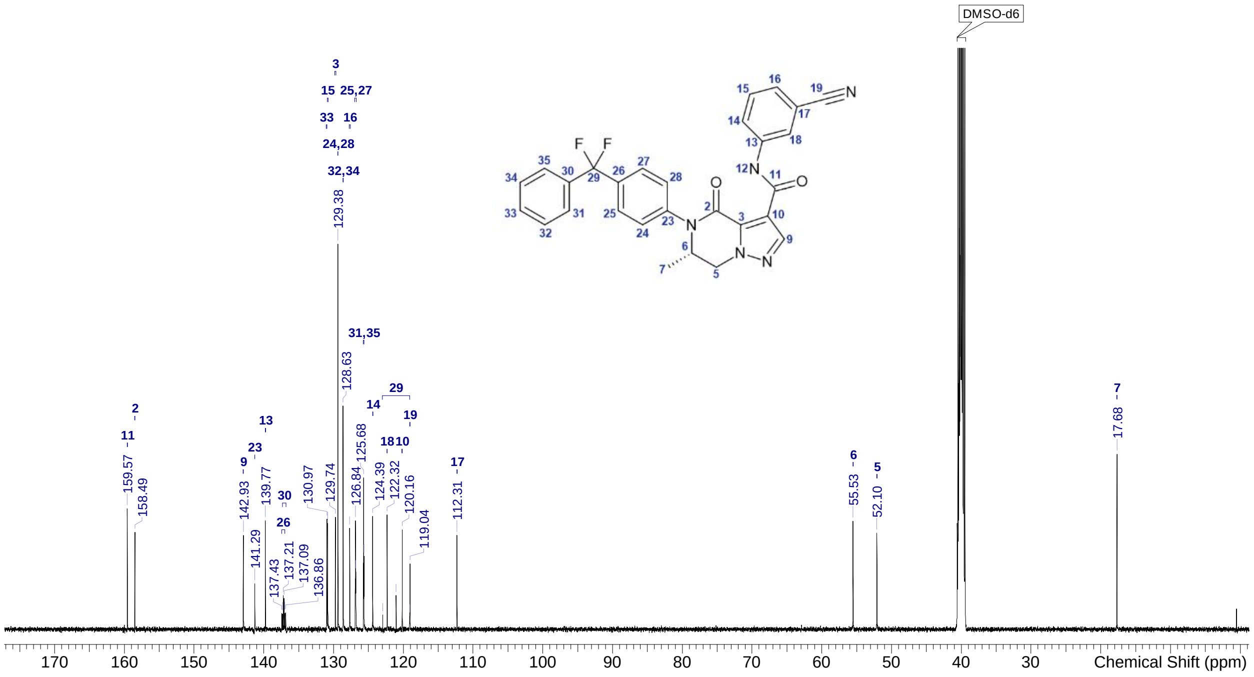

^{13}C NMR (125.76 MHz, DMSO-d_6) δ ppm 159.57 (s, 1 C) 158.49 (s, 1 C) 142.93 (s, 1 C) 141.29 (s, 1 C) 139.77 (s, 1 C) 137.21 (t, *J*=28.04 Hz, 1 C) 137.09 (t, *J*=28.95 Hz, 1 C) 130.97 (s, 1 C) 130.84 (s, 1 C) 129.74 (s, 1 C) 129.38 (s, 2 C) 128.63 (s, 2 C) 127.68 (s, 1 C) 126.84 (t, *J*=5.52 Hz, 2 C) 125.68 (t, *J*=5.52 Hz, 2 C) 124.39 (s, 1 C) 122.32 (s, 1 C) 120.16 (s, 1 C) 121.03 (t, *J*=242.67 Hz, 1 C) 119.04 (s, 1 C) 112.31 (s, 1 C) 55.53 (s, 1 C) 52.10 (s, 1 C) 17.68 (s, 1 C)

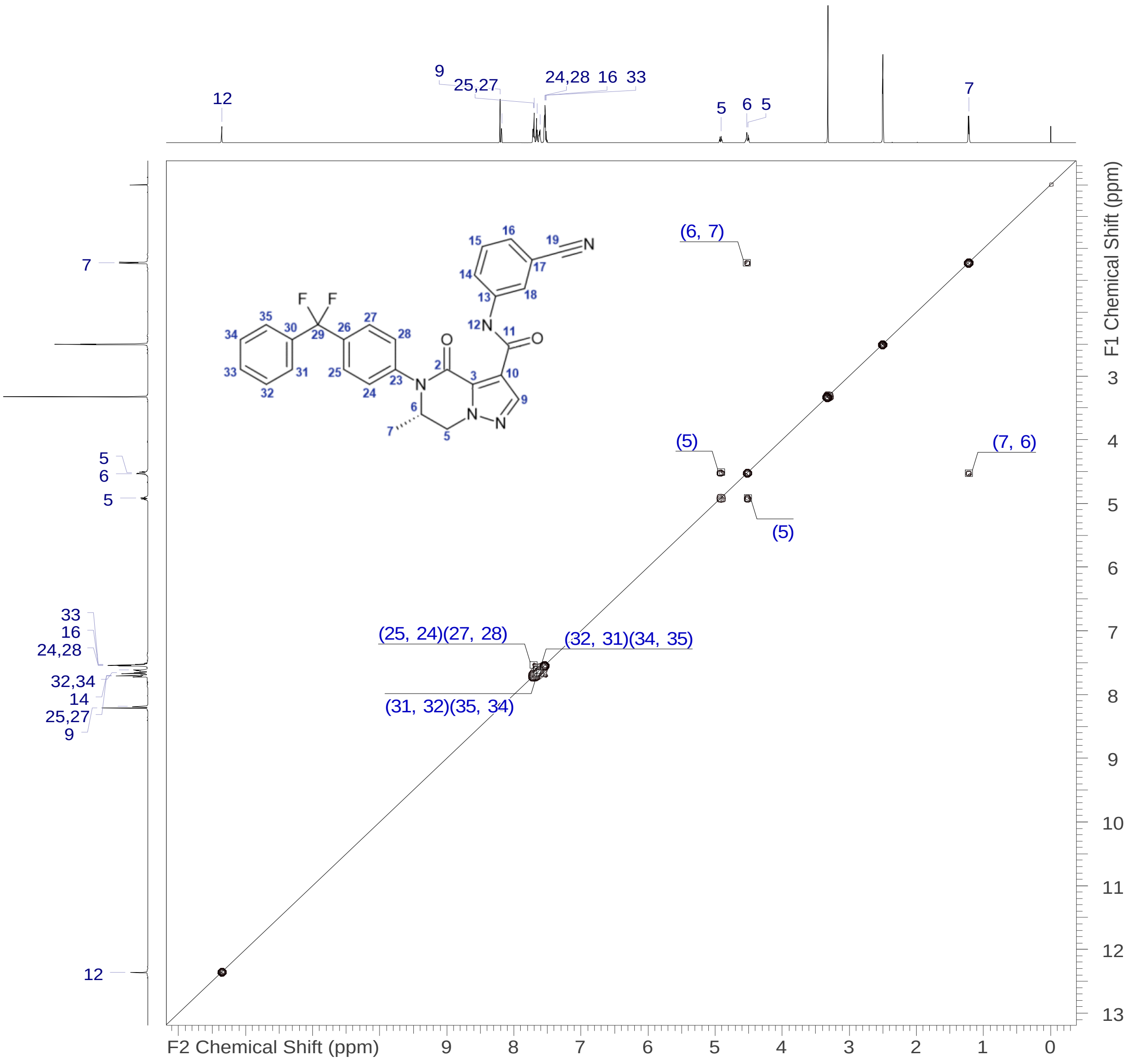

^{1}H-^{1}H COSY correlation spectroscopy NMR (500.13 MHz, DMSO-d_6) experiment. Cross-peaks with assignment in blue are shown indicating through-bond interactions between protons.

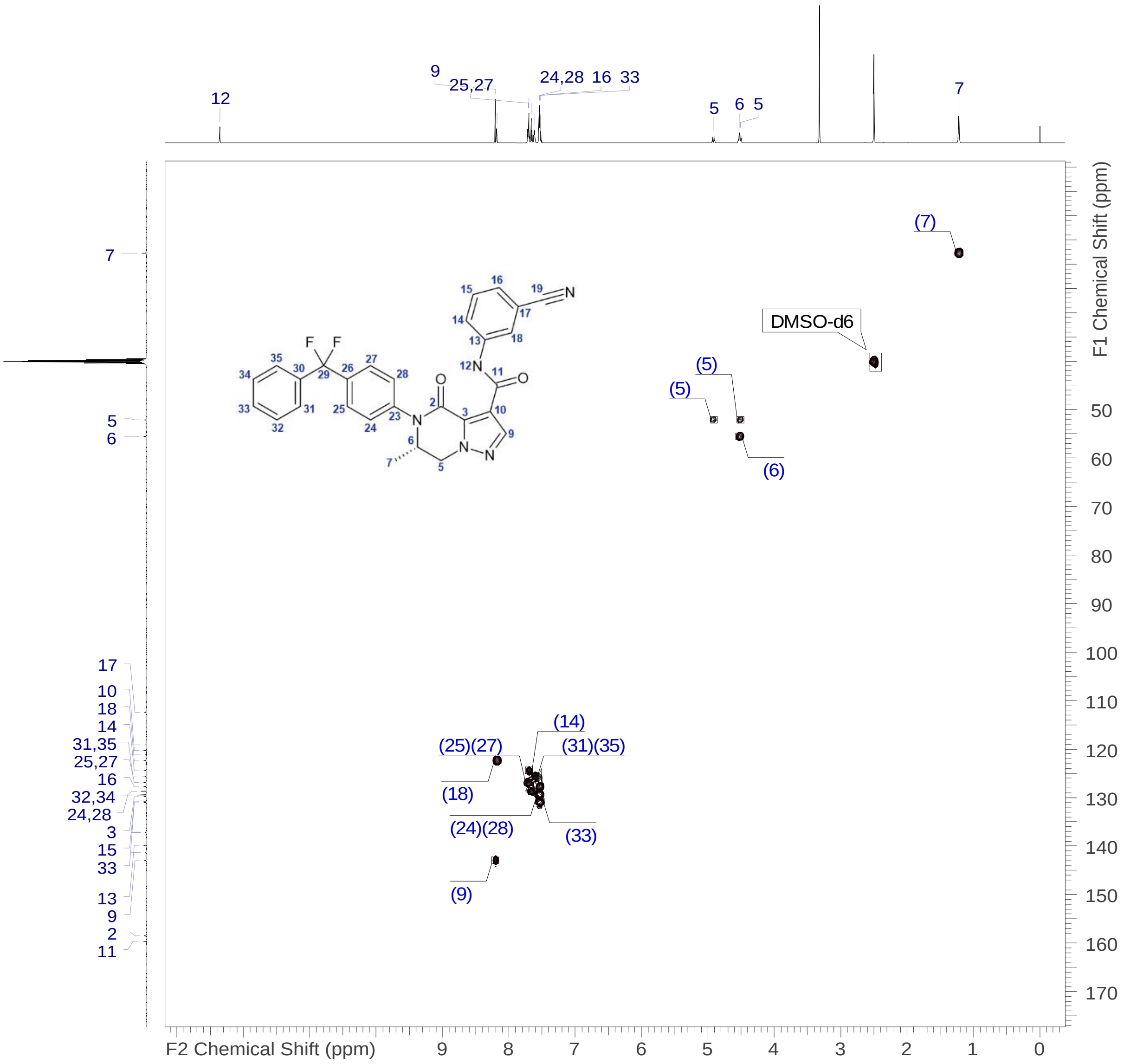

^{1}H-^{13}C HSQC heteronuclear single quantum coherence NMR experiment (^{1}H 500.13 MHz and ^{13}C 125.76 MHz, DMSO-d_6) experiment. Direct correlations between protons and carbon atoms are shown.

Additional cells used for antiviral assays

HeLa-hACE2 cells for the HCoV-229E HCI assay were obtained from Creative Biogene and cultured in DMEM (Gibco) supplemented with 10% v/v heat-inactivated FCS (Biowest), 2 mM alanyl-glutamine (Sigma), 20 µg/mL gentamicin (Gibco), and 0.5 µg/mL puromycin (Gibco).

Huh7 for the HCoV-229E, infectious bronchitis virus (IBV), porcine deltacoronavirus (PDCoV) and mouse hepatitis virus (MHV) assays and HeLa cells for the MHV assay were obtained from ATCC and propagated in DMEM with 4% heat-inactivated FCS with 2 mM alanyl-glutamine, 0.04% gentamycin. Huh7 cells for the Middle East respiratory syndrome coronavirus (MERS-CoV) assay were cultured in DMEM (Gibco) supplemented with 7.87% FBS, 0.89% penicillin/streptomycin, 0.89% L-glutamine and 0.89% non-essential amino acids. Assay medium was DMEM supplemented with 10% FCS, 130 U/mL penicillin, 0.13 mg/mL streptomycin, 20 mM L-glutamine, and 1x non-essential amino acids.

LLC-MK2 cells (Rhesus monkey kidney epithelial cell line; Evotec) for the human coronavirus NL63 (HCoV-NL63) assay were cultured in Eagle's minimum essential medium (EMEM; Sigma) supplemented with 5% v/v FBS, 1% L-glutamine, 1% penicillin/streptomycin, 1% sodium pyruvate, and 1% non-essential amino acids. Assay medium contained only 2% v/v FBS and no sodium pyruvate.

MRC-5 cells (Medical Research Council cell strain 5; Evotec) for the human coronavirus OC43 (HCoV-OC43) assay were cultured in DMEM-high glucose (Gibco) supplemented with 10% v/v FBS, 1% non-essential amino acids, and 1% penicillin/streptomycin. The assay medium contained only 2% v/v FBS.

All cell cultures were checked mycoplasma contamination and found negative.

Viruses

HCoV-229E was obtained from ATCC (VR-740). Virus stocks were obtained after two passages in Huh7 cells, after which stocks were aliquoted, flash-frozen, and stored at -80°C.

IBV (strain Beaudette), PDCoV (strain OH-FD22) and MHV (strain A59 [MHV-2a-FL-Srec] engineered to express firefly luciferase as a reporter) stocks were obtained and used for testing at the University of Utrecht, the Netherlands.

HCoV-NL63 and HCoV-OC43 stocks were obtained and used for testing at Evotec, Toulouse, France.

MERS-CoV (strain Jordan N3) stocks were obtained and used for testing at LUMC, Leiden, The Netherlands.

Broad-spectrum coronavirus antiviral assays

HCoV-229E antiviral assay with high-throughput confocal imaging (HCI) readout

This HCI-based assay was similar to the one in A549-hACE2 cells, but HeLa-hACE2 cells were infected with HCoV-229E at MOI of 0.1[65].

IBV and PDCoV antiviral CPE reduction assay

Huh7 cells were seeded at cell density of 12,000 cells per well in 96-well plates and cultured overnight. Compounds were tested in serial dilutions starting from 30 µM in 3-fold dilution steps. Virus (PDCoV: MOI of 0.1; IBV: MOI of 0.01; HCoV-229E: MOI of 0.01; or mock infection) and compound were added simultaneously; and cultured for 72 h until CPE was clearly visible. At this time point, cell viability was measured. Viability of PDCoV- and IBV-infected cells was determined with Promega Viral ToxGlo™ assay. Viability of HCoV-229E-infected cells was

determined with Promega CellTiter 96™ via a colorimetric assay (MTS assay). Readout was done using a GloMax® Discover Microplate Reader (Promega).

Data were normalized taking the infected cells without treatment and the mock non-infected cells as the bottom and top values of the dose-response curves, respectively. Dose-response curves were fitted using non-linear regression analysis employing a sigmoidal model in Graphpad Prism (version 9.5.0). In each experiment, data were presented as mean ± standard error of the mean of 3 technical replicates.

HCoV-OC43 antiviral CPE reduction assay

For the HCoV-OC43 CPE reduction assay, MRC-5 cells were seeded in black or white 96-well plates (Greiner Bio-One) at a density of 10,000 cells/well and incubated overnight at 37°C. The next day, compound was added to the assay plates in either a 4-fold dilution series or 2-fold dilution series. Sequentially, cells were infected with HCoV-OC43 at a final MOI of 0.3. Plates were then incubated for 6 days at 33°C under 5% CO_2. At this time point, viability of infected cells was determined using the Viral ToxGlo™ assay (Promega) on a BioTek spectrophotometer. Cytotoxicity was evaluated in parallel in treated, uninfected cells in a similar set-up with the same readout. EC_{50} and CC_{50} values were calculated using GraphPad Prism 9.5.0.

HCoV-NL63 CPE reduction assay

LLC-MK2 cells were seeded into black 96-well plates (Greiner Bio-One) in assay medium at a density of 15,000 cells/well and incubated overnight at 37°C. The next day, compound was added in a 2-fold dilution series. Sequentially, cells were infected with HCoV-NL63 at a final MOI of 0.1. Plates were then incubated for 6 days at 34°C under 5% CO_2. Readouts for antiviral activity and toxicity was identical to the procedure for HCoV-OC43 listed above.

MHV antiviral assay

Huh7 cells were seeded in 96-well assay plates at 12,000 cells/well, in 50 µL of high glucose DMEM (with sodium pyruvate and glutaMAX™ supplemented by the vendor) supplemented with 10% v/v FBS and 1% v/v penicillin/streptomycin. The MHV antiviral activity assay was also performed in HeLa cells, which were seeded in 96-well assay plates at 10,000 cells/well, in 50 µL of the same assay medium.

After 24 h incubation, 8-point compound dilution series with a serial dilution factor of 3.4-fold were prepared in assay medium. The cells were inoculated with MHV at an MOI of 0.1. Immediately upon virus addition, the prepared compound dilution series was added to the corresponding wells. Plates were then placed in a humidified incubator for 22–24 h at 37°C. Readout was done using a luminescence readout. Readout was done using a GloMax® Discover Microplate Reader (Promega).

The luminescence intensity was correlated to the dose-dependent effect of the compound as inhibition of viral replication. EC_{50} and EC_{90} values were calculated using GraphPad Prism 9.5.0.

MERS-CoV antiviral CPE reduction assay

Huh7 cells were plated at 10,000 cells/well in 96-well plates and cultured overnight at 37°C. JNJ-9676 was tested in a 2-fold serial dilution. Immediately after compound addition, MERS-CoV (MOI of 0.015) or medium (mock infection) was added. After 48 h incubation at 37°C, the cell viability was determined using a colorimetric MTS assay (Celltiter 96 Aqueous MTS reagent powder, Promega) on an Envision multimode plate reader (Perkin Elmer). Data were normalized by dividing the mean value of the quadruplicates minus the mean background signal (condition without cells) by the mean value of the cell control (condition without virus and compound) minus the mean background signal. EC_{50} and EC_{90} values were calculated using GraphPad Prism 9.5.0.

Time-of-addition assay

15,000 HeLa-hACE2 cells/well were seeded in 96-well plates (Nunclon Delta-Treated, Thermo Fisher Scientific) in DMEM with 10% heat-inactivated FBS and incubated for 24 h at 37°C. On Day 2, the cells were infected with SARS-CoV-2 (B1) (MOI 4) in medium with DMSO (0.05%). The cell control was exposed to medium with the same DMSO concentration. After 1 h incubation at 37°C, the plate was washed four times with pre-warmed medium. After the washes, a sample was taken which was considered the baseline supernatant. At different time points (0, 3, 5 hpi), JNJ-9676 dissolved in medium was added to the infected wells at a final compound concentration of 5 µM. To wells that received compound during infection, the compound was re-administered after the wash. The wells not exposed to compound (cell control and virus control) received medium with the same DMSO concentration (fc 0.05%). Importantly, compound was washed away at different timepoints, i.e., after 1-h compound incubation or at 6 hpi. The cells were incubated at 37°C until 12 hpi. Following the incubation period, medium was collected for each condition and stored at -80°C (supernatant fractions). These supernatant fractions were used for the reinfection experiment (4 replicates).

In the reinfection experiment, Vero E6 cells were seeded at 25,000 cells/well in MEM with 2% heat-inactivated FBS and incubated for 24 h at 37°C. The next day, 10-fold dilutions series were prepared for each supernatant fraction in pre-warmed MEM medium and 50 µl of these virus dilutions was added to the cells. After 72, 96 and 120 h incubation, all the wells were microscopically scored on CPE.

Sequence Alignment

The amino acid sequences for the M protein were downloaded from https://www.ncbi.nlm.nih.gov/ (dated 2023/01/31) and aligned through a pairwise sequence alignment using the Needleman-

Wunsch algorithm[76] through the EMBOSS-Needle tool from EMBL-EBI (https://www.ebi.ac.uk/jdispatcher/psa/emboss_needle). Global alignment was performed, with gap opening penalty of 10, gap extension penalty of 0.2 and the BLOSUM62 as substitution matrix. For the focus on the binding pocket the codon position of the B1 strain was used as a reference. Positions L29, I32, C33, Q36, F37, W55, W58, P59, T61, L87, V88, M91, W92, Y95, F96, S99, F112, N113, P114, E115, T116, and N117 were isolated in the alignment. All visualizations of the alignments were made using Tableau Software.

In vitro resistance selection assay

In vitro resistance selection (IVRS) experiments were performed with SARS-CoV-2 B1, Delta B.1.617.2 and Omicron B.1.1.529-BA.1 under pressure of increasing concentrations of JNJ-9676 in a 96-well plate. A549-hACE2 cells were seeded in assay medium at a density of 5,000 cells/well (3 days of incubation) or 3,000 cells/well (4 days of incubation) in a 96-well plate and were immediately inoculated with SARS-CoV-2 at MOI of 0.01 or 0.02 based on MOI optimization experiments in the presence of compound or DMSO for virus and cell control conditions. Compounds were added in nine 2-fold dilutions starting at a concentration of 4 µM for JNJ-9676. Three replicates were performed per compound. After incubation, every well was scored microscopically for CPE and RT-qPCR was performed regularly as an additional control for the CPE readout. The virus was passaged on new A549-hACE2 compound plates until full infection as determined by CPE scoring was reached for the three replicates. The supernatants of the highest compound concentrations with virus-induced CPE were collected for RNA extraction together with a virus control that was passaged on the plates, and together with the original virus stocks used for infection. These RNA samples were used for the preparation of a library pool for Illumina next generation sequencing, based on the workflow of the NuGen Trio RNASeq™ kit (Tecan

Genomics). FastQ data were analyzed to determine the amino acid changes observed as compared to the reference SARS-CoV-2 sequence (i.e., sequence of the virus inoculation stock) and the frequency of those substitutions. Only samples with an average coverage of more than 1,000 reads per position were proceeded for further bioinformatic analysis. A read frequency threshold of 15% was applied for variant calling. Mutations from compound-resistant viruses were filtered through by comparing to the virus control samples to filter out any potential cell adaptation related mutations.

Generation of site-directed viral mutants

Site-directed mutagenesis in a reverse genetic system and re-culturing in permissive Vero E6 cells were performed to engineer infectious SARS-CoV-2 recombinant clones harboring mutations identified in the SARS-CoV-2 M gene using in vitro resistance selection experiments with JNJ-9676. Full-length SARS-CoV-2 cDNA was assembled from the PCR amplified viral cDNA fragments and the linker fragment into a circular DNA in a single circular polymerase extension reaction (CPER) using a high-fidelity DNA polymerase as described[77]. The primers for CPER reaction are listed in the Supplementary Table 1. Mutations were introduced via site-directed mutagenesis using either Q5® Site-Directed Mutagenesis Kit or Phusion PCR. In brief, the SARS-CoV-2 Belgian strain (B.1) genetic backbone was divided across seven different pUC57 plasmids. These plasmids (pUC57_F1-pUC57_F7) cover the complete SARS-CoV-2 genome. Mutations were introduced in the plasmid that aligned with the region of interest of the virus genome. SDM primers were designed with the NEBaseChanger tool (https://nebasechanger.neb.com/) and inverse PCR was performed using the Q5® Site-Directed Mutagenesis Kit (New England Biolabs, MA, USA) following the manufacturer’s instructions. After DpnI (Invitrogen, MA, USA) digestion of methylated fragments, 5’ ends of PCR products were phosphorylated with

polynucleotide kinase (Invitrogen, MA, USA) and ultimately circularized by in vitro ligation with T4 DNA ligase (Invitrogen, MA, USA). Mutated plasmids were propagated in E. coli DH10B T1R cells, following ampicillin selection of positive clones and validation by Sanger sequencing. The mutated plasmids were used as PCR template in the CPER reaction for generation of the full-length circular site mutant recombinant construct. Only the high frequency accumulated site mutations in the M protein were introduced as single mutations (L29F, A85S, L90W, N117K, P132S, and Q185K). The following top 3 single mutations (W55F, M91K, S99A) were also introduced after resolving the M protein-compound complex cryo-EM structure. For introducing these mutations, a simplified fusion PCR was used by using overlapping primers (Supplementary Table 1) covering the desired mutation followed by the same procedure of CPER reaction.

For recovery of the site-directed mutant virus, 20 µL purified CPER reactions were transfected in BHK-21 cells in 6-well plates using Lipofectamine 3000 reagent (Invitrogen, USA) as per manufacturer's protocol. Seventy-two hours post transfection, supernatants containing viruses were harvested and amplified twice on Vero E6 cells to generate viral stocks. The titer of these 'rescued' infectious clones was determined by CPE-based end-point titration on Vero E6 cells and sequencing of the recombinant virus stocks was performed.

Replication kinetic studies and plaque assay for site-directed mutant viruses

Virus replication kinetics of the recombinant virus strains was assessed in A549-hACE2 cells. Cells were pre-seeded at 12,500 cells/well into 96-well plates. 24 h after seeding, cells were infected with SARS-CoV-2 wild-type (WT), recombinant wild type and M protein mutated virus: SARS-CoV-2-recombWT, L29F, A85S, L90W, N117K, P132S, Q185K, W55F, M91K, S99A, N117K+P132S, N117K+L29F+A85S and P132S+L138I+S173P+Q185K, at an MOI of 0.1. Viruses were washed away after 1 h incubation. Viral supernatants were collected for RT-qPCR

analysis and cells were fixed for staining of viral spike protein and dsRNA at indicated timepoints (1, 4, 8, 24, 48, 72 hpi). The kinetics profile for various recombinant strains of SARS-CoV-2 in aspects of dsRNA, spike and viral RNA abundancy throughout the duration of the experiment were compared with WT recombinant virus. Plaque assay of the recombinant virus strains was performed in Vero E6 cells. Cells were pre-seeded at 25000 cells/well into 12-well plates. 24h after seeding, cells were infected SARS-CoV-2 wild-type (WT), recombinant wild type M protein virus: SARS-CoV-2-recombWT, L29F, A85S, L90W, N117K, P132S, Q185K, W55F, M91K, S99A, N117K+P132S, N117K+L29F+A85S and P132S+L138I+S173P+Q185K, with 250 µL corresponding serial dilution (=inoculum). Cells were incubated with virus for 1 hour at 37°C. In the meantime, the agarose overlay was prepared (50% of 1% UltraPure™ Low Melting Point Agarose (ThermoFisher) + 50% of 2X medium (Gibco™ MEM (Temin's modification) (2X), 4% fetal bovine serum (Biowest), and 0.08% gentamicine (Gibco)). After 1 hour of incubation the inoculum was removed from the wells and 1 mL of the agarose overlay was added to each well. Once the agarose overlay solidified, plates were placed upside down in the incubator for 3 days at 37°C, 5% CO_2. Cells were fixed with 2 ml/well of 4% formaldehyde (Polysciences Inc.) at 3 dpi and the plates were kept 2 hours under the laminar flow for soaking. Afterwards the agarose overlay and formaldehyde was removed and the plates were washed with water. Next, pre-made 0.1% crystal violet (Sigma) was added to the plates and incubated for 10 minutes. After staining, the plates were washed with PBS. The plaques size was visually assessed and compared between wild-type viruses and recombinant mutant viruses.

Post-exposure Syrian golden hamster model (Evotec)

Housing conditions and experimental procedures were performed as described in project APAFIS#31467-2021041618563995 as approved by the ethics committee of Evotec (France) which is licensed under number E31555059 and LA1100119.

Statistical power analysis as well as the limitations of the study size warranted 8 animals per group to obtain statistical significance in Syrian golden hamster studies. Upon arrival, animals were randomly assigned in groups. No blinding was performed during the experiment. Female Syrian golden hamsters (Janvier Laboratories) of 8–10 weeks old were anesthetized by isoflurane inhalation and inoculated intranasally with 110 µL of PBS containing 2.2×10^5 PFU of SARS-CoV-2 (USA-WA1/2020) on day 0. Animals were treated orally starting 1h before or 10h after infection and continued to be dosed twice daily at 8h intervals with vehicle or JNJ-9676 (75 mg/kg/dose in 100% PEG400). On day 4 pi, hamsters were euthanized by CO_2 inhalation. Whole right lungs were homogenized in PBS (ratio of 0.5g of lung per 1 mL PBS) using the gentleMACS™ dissociator (Miltenyi) and C tubes. Viral RNA and infectious virus levels were quantified in the lung homogenate supernatant by RT-qPCR and plaque assay, respectively (Extended Data Fig. 5d-e).

RNA extraction was performed using the Maxwell RSC simply RNA tissue kit (Promega). Briefly, 40µL of samples homogenate (20mg of tissue) was mixed with 200µL of 1-thioglycerol/ homogenization solution. 200µL of Lysis buffer was added to the tissue homogenate and the 400µL was transferred to the Maxwell RSC simply RNA Tissue cartridge. RNA extraction was performed using the Maxwell instrument following guidelines and programs provided by the manufacturer (Promega). The RNA levels were quantified using a standard range, the TaqMan Fast Virus 1-step Master Mix (ThermoFisher #4444434) and the following primers and probe: Fwd: GACCCCAAAATCAGCGAAAT, Rev: TCTGGTTACTGCCAGTTGAATCTG, Probe

FAM: ACCCCGCATTACGTTTGGTGGACC. The standard range was prepared by 10-fold serial dilution of qPCR Control RNA from inactivated SARS-CoV-2 (strain USA-WA1/2020, BEI Resources #NR-52347).

For plaque assay, lung homogenate supernatant was serially diluted 10-fold in infection medium (DMEM glutamax medium supplemented with 2% fetal bovine serum, 1% Penicillin/streptomycin (10000U/mL) and 0.1% Hepes (1M)). Next, 100µL of each dilution was added to confluent Vero E6 cells in 24-well plates and incubated at 37°C, 5% CO_2. After 1h of incubation, 0.5mL of MEM: methylcellulose mixture (2X MEM + 4% FBS with 2% methylcellulose in a 1:1 ratio) was added to each well and plates were further incubated at 37°C, 5% CO_2 for 3 days. On day 4, plaques were detected by gently removing methylcellulose overlays and fixing cells with 4% PFA in PBS for 30 min at RT. Finally, 0.05% crystal violet (w/v) solution was added to the cells for 10 min at RT. The plaques were counted for each dilution and the viral titer was determined and expressed in PFU/g of tissue.

Histopathology was performed on formalin-inflated left lung lobes, which were embedded in paraffin, sectioned (5 µm), and stained with hematoxylin-eosin (H.E.) (Extended Data Fig. 5f). Following H.E. staining, histopathological examination was performed on the lungs. The microscopic findings (graded as 1: minimal histological change, 2: slight/mild, 3: moderate, 4: marked, and 5: severe/massive histological change) included: alveolar edema, alveolar hemorrhage, alveolar infiltrate, alveolar/interstitial inflammation, bronchitis/bronchiolitis, perivascular cuffing, thickening of alveolar septa, and type II pneumocyte hyperplasia. Additionally, paraffin sections were stained immunohistochemically for SARS-CoV-2 nucleoprotein (with SARS-CoV/SARS-CoV-2 nucleoprotein/nucleocapsid antibody, rabbit

polyclonal antibody; SinoBiological, 40143-T62; 1:6,000 dilution followed by detection with an anti-rabbit HQ/anti HQ-HRP detection system; Roche, 07017812001/07017936001) (Extended Data Fig. 5g). These sections were also semi-quantitatively evaluated for the number of SARS-CoV-2 nucleoprotein immunoreactive cells in lung parenchyma (positive pneumocytes and macrophages) and in bronchi/bronchioles (positive epithelial cells). Positivity for SARS-CoV-2 nucleoprotein was scored as 1: minimal, 2: mild, 3: moderate, 4: marked, and 5: massive numbers of positive cells.

The statistical analysis was performed as described above.

Supplementary Tables

Supplementary Table 1. List of primers used for CPER and site-directed mutagenesis

Primers	Sequence (5'-3')
CPER_F1_F	5'-TCCCAGGTAACAAACCAACCAACTTTCG-3'
CPER_F1_R	5'-GTAGGCTAAGATAAGTGCACAAAAGTTAGCAG-3'
CPER_F2_F	5'-GTGCACTTATCTTAGCCTACTGTAATAAGACAG-3'
CPER_F2_R	5'-GAACCCTTAATAGTGAAATTGGGCCTCATAGCAC-3'
CPER_F3_F	5'-CCCAATTTCACTATTAAGGGTTCATTCCTTAATGG-3'
CPER_F3_R	5'-CATCCTGATTATGTACAACACCTAGCTCTCTGAAGTGG-3'
CPER_F4_F	5'-GGTGTTGTACATAATCAGGATGTAAACTTACATAGC-3'
CPER_F4_R	5'-CATATTCTGAGCCCTGTGATGAATCAACAGTTTG-3'
CPER_F5_F	5'-ATCACAGGGCTCAGAATATGAC-3'
CPER_F5_R	5'-GTTGTTTTCTCTAATTATAAGTCTACCTTTACTAAGAAG-3'
CPER_F6_F	5'-GGTAGACTTATAATTAGAGAAAACAACAGAG-3'
CPER_F6_R	5'-GTTACAGTTCCAATTGTGAAGATTCTCATAAACAAATCC-3'
CPER_F7_F	5'-CTTCACAATTGGAACTGTAACTTTGAAGC-3'
CPER_F7_R	5'-GTCATTCTCCTAAGAAGCTATTAAAATCACATGGGG-3'
CPER_linker_F	5'-TAGCTTCTTAGGAGAATGACAAAAAAAAAAAAAAAAAAAAAAAAAAAAAAAGGGTCGGCATGGC-3'
CPER_linker_R	5'-GGTTGGTTTGTTACCTGGGAAGGTATAAACCTTTAATCGGTTCACTAAACGAGCTCTGCT-3'
F7_M_L29F_F	5'-TTTCCTATTCtTTACATGGATTTG-3'
F7_M_L29F_R	5'-CCTATTACTAGGTTCCATTG-3'
F7_M_A85S_F	5'-TATCGCAATGtCTTGTCTTGTAGG-3'
F7_M_A85S_R	5'-GCAATTCCACCGGTGATC-3'
F7_M_N117K_F	5'-CAGAAACTAAgATTCTTCTCAAC-3'
F7_M_N117K_R	5'-GATTGAATGACCACATGG-3'
F7_M_P132S_F	5'-TCTGACCAGAtCGCTTCTAGAAAG-3'
F7_M_P132S_R	5'-ATAGTGCCATGGAGTGGC-3'
F7_M_L90W_F	5'-CTTGTAGGCTgGATGTGGCTC-3'
F7_M_L90W_R	5'-ACAAGCCATTGCGATAGC-3'
F7_M_S173P _F	5'-TGTTGCTACAcCACGAACGCT-3'
F7_M_S173P_R	5'-GTGATTTCTTTAGGCAGGTC-3'
F7_M_Q185K_F	5'-GGGAGCTTCGaAGCGTGTAGC-3'
F7_M_Q185K_R	5'-AATTTGTAATAAGAAAGCGTTCGTGATGTAG-3'
F7_M_W55F-F	5'-TTTCCTCTTTCTGTTATGGCCAGTAACT-3'
F7_M_W55F-R	5'-GCCATAACAGAAAGAGGAAAATTAAC-3'
F7_M_M91K-F	5'-AGGCTTGAAGTGGCTCAGCTACTTCA-3'
F7_M_M91K-R	5'-GCTGAGCCACTTCAAGCCTACAAGAC-3'
F7_M_S99A-F	5'-ATTGCTGCTTTCAGACTGTTTGCGCGTAC-3'
F7_M_S99A-R	5'-ACAGTCTGAACGAAGCAATGAAGTAGCTGA-3'
F7_M_A98D-F	5'-TACTTCATTGATTCTTTCAGACTGTTTG-3'
F7_M_A98D-R	5'-GCTGAGCCACATCAAGCC-3'

References

65 Doijen, J. *et al.* A flexible, image-based, high-throughput platform encompassing in-depth cell profiling to identify broad-spectrum coronavirus antivirals with limited off-target effects. *Antiviral Res.*, 105789 (2023).

76 Needleman, S. B. & Wunsch, C. D. A general method applicable to the search for similarities in the amino acid sequence of two proteins. *J. Mol. Biol.* **48**, 443-453 (1970).

77 Amarilla, A. A. *et al.* A versatile reverse genetics platform for SARS-CoV-2 and other positive-strand RNA viruses. *Nat. Commun.* **12**, 3431 (2021).
