# Supplementary material for: A small-molecule SARS-CoV-2 inhibitor targeting the membrane protein
Source: Nature. 2025 Mar 26;640(8058):506–13. doi: 10.1038/s41586-025-08651-6 (PMC11981937; doi:10.1038/s41586-025-08651-6)
Supplement: Supplementary file 2 — Reporting Summary [file 41586_2025_8651_MOESM2_ESM.pdf]

nature portfolio

Corresponding author(s): Marnix Van Loock, Anil Koul, Ruxandra Draghia-Akli

Last updated by author(s): Oct 29, 2024

Reporting Summary

Nature Portfolio wishes to improve the reproducibility of the work that we publish. This form provides structure for consistency and transparency in reporting. For further information on Nature Portfolio policies, see our Editorial Policies and the Editorial Policy Checklist.

Statistics

For all statistical analyses, confirm that the following items are present in the figure legend, table legend, main text, or Methods section.

n/a	Confirmed	
☐	☒	The exact sample size (*n*) for each experimental group/condition, given as a discrete number and unit of measurement
☐	☒	A statement on whether measurements were taken from distinct samples or whether the same sample was measured repeatedly
☐	☒	The statistical test(s) used AND whether they are one- or two-sided<br>*Only common tests should be described solely by name; describe more complex techniques in the Methods section.*
☒	☐	A description of all covariates tested
☐	☒	A description of any assumptions or corrections, such as tests of normality and adjustment for multiple comparisons
☐	☒	A full description of the statistical parameters including central tendency (e.g. means) or other basic estimates (e.g. regression coefficient) AND variation (e.g. standard deviation) or associated estimates of uncertainty (e.g. confidence intervals)
☐	☒	For null hypothesis testing, the test statistic (e.g. *F*, *t*, *r*) with confidence intervals, effect sizes, degrees of freedom and *P* value noted<br>*Give P values as exact values whenever suitable.*
☒	☐	For Bayesian analysis, information on the choice of priors and Markov chain Monte Carlo settings
☒	☐	For hierarchical and complex designs, identification of the appropriate level for tests and full reporting of outcomes
☒	☐	Estimates of effect sizes (e.g. Cohen's *d*, Pearson's *r*), indicating how they were calculated

Our web collection on statistics for biologists contains articles on many of the points above.

Software and code

Policy information about availability of computer code

Data collection	Antiviral activity against SARS-CoV, SARS-CoV-2 and 229E was done by high content imaging in A549-hACE2 cells on a Cell Voyager 8000 (Yokogawa) confocal microscope whereas antiviral activity VeroE6-eGFP using HCI was done on a Arrayscan XTI (Thermofisher). MTS assays to assess toxicity were read out on a Spark plate reader (Tecan).<br>Antiviral activity against zoonotic viruses was measured using NanoGlo on a Glomax plate reader (Promega).<br>Antiviral activity against IBV, MHV and PDCoV was done using a GloMax® Discover Microplate Reader (Promega).<br>Antiviral activity against OC43 and NL63 was measured on a BioTek spectrophotometer.<br>Antiviral activity against MERS was measured on an Envision multimode plate reader (Perkin Elmer).<br>All RNA extractions were automated on a MagNA Pure instrument (Roche) and RT-qPCR readouts were obtained on a LightCycler 480 real-time PCR instrument (Roche).<br>For viral yield studies, RNA extraction and RT-qPCR results were obtained as listed above. The parallel toxicity readout was done on a Viewlux instrument (PerkinElmer).<br>In ALI cultures, antiviral activity was measured by RT-qPCR as mentioned above. TEER toxicity measurements on ALI cultures were performed using the EVOM3 (World Precision Instruments).<br>NanoDSF measurements were taken on a Prometheus NT.Plex instrument (NanoTemper Technologies).<br>For ASMS, all liquid chromatography-mass spectrometry (LC-MS) analyses were performed on an 1290 Infinity II uHPLC system (Agilent) coupled to a 6545XT qTOF (Agilent).<br>Cryo-EM data collection was automated on a 200 kV Glacios™ microscope (Thermo Scientific).Micrographs were taken at 105,000X magnification using a Facon4 detector (Gatan) in counting mode.<br>1H NMR spectra were recorded on a Bruker DPX-400 spectrometer.<br>1H-13C HMBC NMR spectra were recorded on a Bruker Avance-500 spectrometer.

Data analysis

All High content imaging analysis was done in Phaedra HCI analysis software (version 1.0.10.202309011029).
All antiviral data (EC50/90, CC50) was processed using Graphpad Prism (version 8 or 9).
Antiviral data in ALI cultures was processed in LightCycler software (Roche) and Graphpad Prism (version 8). Toxicity CC50 values were calculated in Graphpad Prism (version 8).
NanoDSF data were analyzed with PR. ThermControl v2.1.6 (NanoTemper Technologies).
ASMS data processing was performed using Agilent MassHunter Qualitative Analysis (v 10.0).
CryoEM structure representations were generated using Pymol (v2.0) and Chimera (v1.17.3).Cryo-EM data collection and image quality were monitored using cryoSPARC Live v3.2. Image. Local resolution was determined using ResMap. For the M/Fab-B complex model building, the M protein was manually built using COOT. The Fab-B was fitted into the 3D map using Chimera and then further refined manually with COOT followed by real-space refinement in Phenix.
The data was processed using the Bruker TOPSPIN program v4.1, and 1H and 13C chemical shifts were analyzed using ACD/Spectrus software 2023 v1.1.
All statistical analyses for in vivo experiments were performed in GraphPad Prism (version 9) and validated using R (version 3.6.1).
The amino acid sequences for the M protein were downloaded from https://www.ncbi.nlm.nih.gov/ (dated 2023/01/31) and aligned through a pairwise sequence alignment using the Needleman-Wunsch algorithm through the EMBOSS-Needle tool from EMBL-EBI (https://www.ebi.ac.uk/jdispatcher/psa/emboss_needle). All visualizations of the sequence alignments were made using Tableau Software (online version).
Graphs and figures were generated using Microsoft PowerPoint (Version 2308 Build 16731.20460), GraphPad Prism (v8 and 9), BioRender (free version, in vivo work), PyMOL Molecular Graphics System (Version 2.0), Chimera (version 1.17.3), CryoSparc (version 4.4.1), 3D-FSC (version 1.0), Grace (version 5.1.25) and Image Lab (version 6.0.1); the software is made available by Janssen Pharmaceutica NV.

For manuscripts utilizing custom algorithms or software that are central to the research but not yet described in published literature, software must be made available to editors and reviewers. We strongly encourage code deposition in a community repository (e.g. GitHub). See the Nature Portfolio guidelines for submitting code & software for further information.

Data

Policy information about availability of data

All manuscripts must include a data availability statement. This statement should provide the following information, where applicable:

- Accession codes, unique identifiers, or web links for publicly available datasets
- A description of any restrictions on data availability
- For clinical datasets or third party data, please ensure that the statement adheres to our policy

All data supporting the findings of this study are available within the article and all accession codes are provided in the manuscript.
Cryo-EM maps have been deposited in the Electron Microscopy Data Bank (accession code: EMD-43745), while the atomic coordinates of the M/Fab-B complex structures have been deposited in the Protein Data Bank (accession code: 8W2E). The PDB accession codes for the M/Fab-B complex short-form is 7VG and for the M/Fab-E long-form is 7VGR.
No cropped images of western blots are shown, the uncropped images of the western blots are presented in Extended Data Fig. 2g.

Research involving human participants, their data, or biological material

Policy information about studies with human participants or human data. See also policy information about sex, gender (identity/presentation), and sexual orientation and race, ethnicity and racism.

Reporting on sex and gender: No human participants were used in this study.

Reporting on race, ethnicity, or other socially relevant groupings: No human participants were used in this study.

Population characteristics: No human participants were used in this study.

Recruitment: No human participants were used in this study.

Ethics oversight: No human participants were used in this study.

Note that full information on the approval of the study protocol must also be provided in the manuscript.

Field-specific reporting

Please select the one below that is the best fit for your research. If you are not sure, read the appropriate sections before making your selection.

☒ Life sciences ☐ Behavioural & social sciences ☐ Ecological, evolutionary & environmental sciences

For a reference copy of the document with all sections, see nature.com/documents/nr-reporting-summary-flat.pdf

Life sciences study design

All studies must disclose on these points even when the disclosure is negative.

Sample size	For almost all antiviral in vitro studies, three or more independent experiments (using multiple technical replicates) were performed (Fig1b, Extended data table 1). Whenever possible, we strived to obtain data from three independent experiments, a common standard for biological experiments which allows to identify outliers or anomalies in the data. No formal size calculation was performed for in vitro experiments. In our analysis of the spectrum against which JNJ-9676 is active, some exceptions to the n=3 rule were made. Either because, in case of antiviral experiments with animal coronaviruses, the materials are very scarce and results were fully in line with those obtained with human coronaviruses. Or, in case of human coronaviruses other than SARS-CoV-2 or SARS-CoV, because antiviral activity was limited or absent and thus irrelevant for future human treatment. These data were merely used to showcase the spectrum against which JNJ-9676 acts.<br>In the translational ALI model, three independent experiments were run to account for potential variation (Fig1c).<br>Resistant selection experiments with the compound were obtained from three different (at the time) variants of concern of SARS-CoV-2 and includes analysis of multiple passages (Fig1d,e; Extended data table 2; Extended data fig1d).<br>Three independent experiments were run with the site directed mutants to obtain EC50 fold changes (Fig1f). Fitness experiments were carried out in three independent experiments (8 technical replicates per experiment)(Extended data fig1g).<br>One representative experiment is shown for the ASMS readout, three technical replicates are plotted (Fig1g).<br>NanoDSF was carried out with the compound in three independent experiments (Fig1h).<br>Time of addition studies were carried out multiple times, a representative experiment is shown with three technical replicates (Extended data fig1b).<br>Given the elaborate evidence and the high labor intensity of these assays; this number of replicates was considered sufficient.<br>Statistical power analysis as well the limitations of the study size warrented 5 animals per group to obtain statistical significance in in vivo Syrian golden hamster studies.
Data exclusions	No data was excluded from any of the studies reported in this paper.
Replication	Three or more independent experiments (either in duplicate or triplicate) were performed for almost all in vitro experiments and at least two independent experiments for almost all in vivo studies. All attempts at replication were consistent and reflect the intra and inter variability.
Randomization	Allocation of hamsters to experimental groups was performed randomly. For the in vitro experiments performed in this study, randomization was not relevant as no allocation to experimental treatment groups is required. Reference compounds and proper controls were taken along to assess consistency over time. We received consistent results over time with repeats performed on different days and by different people.
Blinding	For both the in vivo and in vitro experiments performed in the study, blinding was not applicable as no experimental treatment groups were used where the quality of the outcome could be influenced.

Reporting for specific materials, systems and methods

We require information from authors about some types of materials, experimental systems and methods used in many studies. Here, indicate whether each material, system or method listed is relevant to your study. If you are not sure if a list item applies to your research, read the appropriate section before selecting a response.

Materials & experimental systems

n/a	Involved in the study	
☐	☒	Antibodies
☐	☒	Eukaryotic cell lines
☒	☐	Palaeontology and archaeology
☐	☒	Animals and other organisms
☒	☐	Clinical data
☒	☐	Dual use research of concern
☒	☐	Plants

Methods

n/a	Involved in the study	
☒	☐	ChIP-seq
☒	☐	Flow cytometry
☒	☐	MRI-based neuroimaging

Antibodies

Antibodies used	SARS-CoV/SARS-CoV-2 nucleoprotein/nucleocapsid antibody, rabbit polyclonal antibody (Sino Biological, 40143-T62); primary anti-

Antibodies used	spike S1 monoclonal antibody (Recombinant, expressed from Hek293 cells; rabbit) (Sino Biological, cat. 40150-R007, Houston, TX, USA); primary anti-double-stranded RNA (dsRNA) monoclonal antibody J2 (mouse) (SCICONS, cat. 10010500, Jena Bioscience, Jena, Germany); secondary goat anti-mouse Polyclonal immunoglobulin G (IgG) secondary antibody, conjugated to Alexa Fluor 488 (goat anti-mouse) (cat. A11001, Invitrogen, Waltham, MA, USA); secondary goat anti-rabbit Polyclonal immunoglobulin G (IgG) secondary antibody, conjugated to Alexa Fluor 568 (cat. A11036, Invitrogen, Waltham, MA, USA), HQ Anti-Rabbit (cat. 07017812001, Roche), Anti-HQ HRP (cat. 07017936001, Roche).
Validation	All antibodies were obtained from commercial sources.<br>The use of SARS-CoV/SARS-CoV-2 nucleoprotein/nucleocapsid antibody, rabbit polyclonal antibody (Sino Biological) was described here PMID: 38920116.<br>The use of SARS-CoV/SARS-CoV-2 spike monoclonal antibody (Sino Biological); J2 mouse anti-dsRNA monoclonal antibody (Scicons); goat anti-rabbit polyclonal Ab conjugated to Alexa Fluor 5681 (Invitrogen); goat anti-mouse IgM polyclonal Ab conjugated to Alexa Fluor 488 (Invitrogen) was described here PMID: 38158129.

Eukaryotic cell lines

Policy information about cell lines and Sex and Gender in Research

Cell line source(s)	Human epithelial cell line A549 stably expressing hACE2 (A549-hACE2) were obtained from InvivoGen (San Diego, USA) or from the American Type Culture Collection (ATCC; # CCL-185).<br>VeroE6-eGFP were cloned and validated at Tibotec/Janssen Pharmaceutica NV (Beerse, Belgium).<br>Pooled donor nasal epithelial cells grown in air-liquid interface (ALI) format were obtained from Epithelix as a fully differentiated culture (Plan-les-Ouates, Switserland).<br>Hela cells were obtained from ATCC.<br>HeLa-hACE2 cells were obtained from Creative Biogene (New York, USA).<br>Huh7 were obtained from ATCC.<br>LLC-MK2 cells were obtained from Evotec (Toulouse, France).<br>MRC-5 cells were obtained from Evotec (Toulouse, France).
Authentication	Cell lines were not authenticated.
Mycoplasma contamination	All cell lines tested negative for mycoplasma contamination.
Commonly misidentified lines<br>(See ICLAC register)	None of the commonly misidentified cell lines were used.

Animals and other research organisms

Policy information about studies involving animals; ARRIVE guidelines recommended for reporting animal research, and Sex and Gender in Research

Laboratory animals	In pre-exposure studies, female Syrian golden hamsters of 6–8 weeks were used purchased from a Janvier Laboratories. In post-exposure studies, male Syrian golden hamsters of 6–8 weeks were used purchased from a Janvier Laboratories.
Wild animals	No wild animals were used in this study.
Reporting on sex	Findings do not apply to one sex only.
Field-collected samples	No field-collected samples were used in the study.
Ethics oversight	Pre-exposure experiments were performed at KU Leuven. Housing conditions and experimental procedures were performed as described in project 062/2020 as approved by the ethics committee of KU Leuven (Belgium) which is licensed under number LA1210186.<br>Post-exposure experiments happened either at Evotec or in house at J&J Innovative Medicine. Housing conditions and experimental procedures were performed as described in project APAFIS#31467-2021041618563995 v3 and Proj 129-Proc 786 as approved by the ethics committee of Evotec (France) and Johnson&Johnson Innovative Medicine (Belgium) which are licensed under number E31555059 and LA1100119, respectively.

Note that full information on the approval of the study protocol must also be provided in the manuscript.

Plants

Seed stocks	No plants were used in this study.
Novel plant genotypes	No plants were used in this study.
Authentication	No plants were used in this study.
