# Supplementary figures and images for: A small-molecule SARS-CoV-2 inhibitor targeting the membrane protein
Source: Nature. 2025 Mar 26;640(8058):506–13. doi: 10.1038/s41586-025-08651-6 (PMC11981937; doi:10.1038/s41586-025-08651-6)

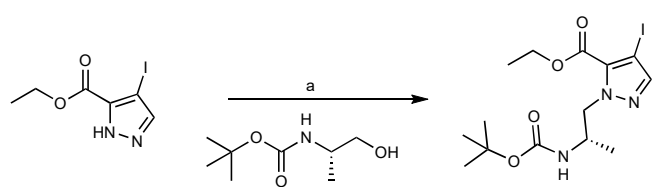

Supplement: Supplementary file 4 — Synthesis procedure of JNJ-9676_reaction (a–e) and Synthesis scheme of JNJ-9676. [file 41586_2025_8651_MOESM4_ESM.zip › 2024-02-03252B-s4/2024-02-03252B-SI_Synthesis procedure of JNJ-9676_reaction a.pdf]

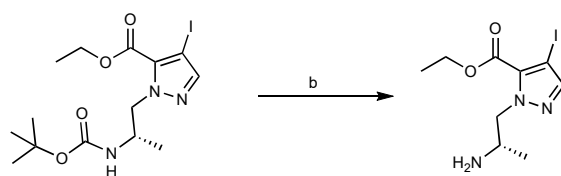

Supplement: Supplementary file 4 — Synthesis procedure of JNJ-9676_reaction (a–e) and Synthesis scheme of JNJ-9676. [file 41586_2025_8651_MOESM4_ESM.zip › 2024-02-03252B-s4/2024-02-03252B-SI_Synthesis procedure of JNJ-9676_reaction b.pdf]

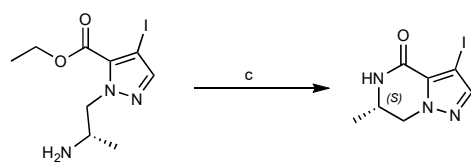

Supplement: Supplementary file 4 — Synthesis procedure of JNJ-9676_reaction (a–e) and Synthesis scheme of JNJ-9676. [file 41586_2025_8651_MOESM4_ESM.zip › 2024-02-03252B-s4/2024-02-03252B-SI_Synthesis procedure of JNJ-9676_reaction c.pdf]

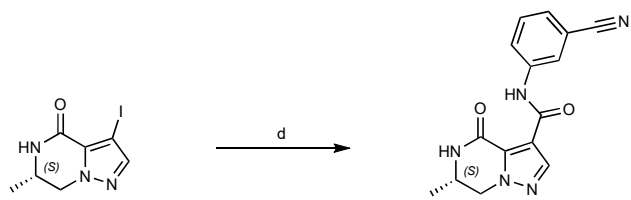

Supplement: Supplementary file 4 — Synthesis procedure of JNJ-9676_reaction (a–e) and Synthesis scheme of JNJ-9676. [file 41586_2025_8651_MOESM4_ESM.zip › 2024-02-03252B-s4/2024-02-03252B-SI_Synthesis procedure of JNJ-9676_reaction d.pdf]

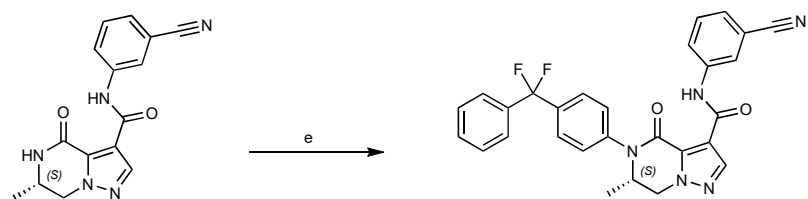

Supplement: Supplementary file 4 — Synthesis procedure of JNJ-9676_reaction (a–e) and Synthesis scheme of JNJ-9676. [file 41586_2025_8651_MOESM4_ESM.zip › 2024-02-03252B-s4/2024-02-03252B-SI_Synthesis procedure of JNJ-9676_reaction e.pdf]

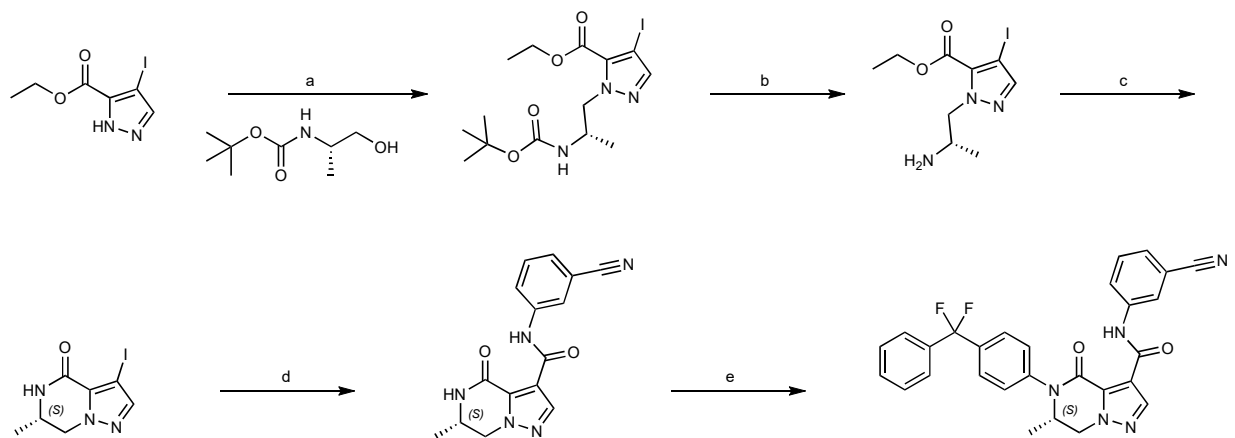

Supplement: Supplementary file 4 — Synthesis procedure of JNJ-9676_reaction (a–e) and Synthesis scheme of JNJ-9676. [file 41586_2025_8651_MOESM4_ESM.zip › 2024-02-03252B-s4/2024-02-03252B-SI_Synthesis scheme of JNJ-9676.pdf]
